# Intersectoral collaboration for people-centred mental health care in Timor-Leste: a mixed-methods study using qualitative and social network analysis

**DOI:** 10.1186/s13033-019-0328-1

**Published:** 2019-11-16

**Authors:** Teresa Hall, Ritsuko Kakuma, Lisa Palmer, Harry Minas, João Martins, Greg Armstrong

**Affiliations:** 1 Nossal Institute for Global Health, University of Melbourne, 333 Exhibition St, Melbourne, VIC 3004 Australia; 2 Centre for Global Mental Health, London School of Hygiene and Tropical Medicine, London, UK; 3 Centre for Mental Health, University of Melbourne, Melbourne, Australia; 4 School of Geography, University of Melbourne, Melbourne, Australia; 5 Faculty of Medicine and Health Sciences, National University of Timor-Leste, Dili, Timor-Leste

**Keywords:** Intersectoral collaboration, Governance, Global mental health, Timor-Leste, Asia Pacific

## Abstract

**Background:**

Intersectoral collaboration is fundamental to the provision of people-centred mental health care, yet there is a dearth of research about how this strategy operates within mental health systems in low- and middle-income countries. This is problematic given the known attitudinal, structural and resource barriers to intersectoral collaboration in high-income country mental health systems. This study was conducted to investigate intersectoral collaboration for people-centred mental health care in Timor-Leste, a South-East Asian country in the process of strengthening its mental health system.

**Methods:**

This study employed a mixed-methods convergent design. Qualitative data elicited from in-depth interviews with 85 key stakeholders and document review were complemented with quantitative social network analysis to assess understandings of, the strength and structure of intersectoral collaboration in the Timorese mental health system.

**Results:**

There was consensus among stakeholder groups that intersectoral collaboration for mental health is important in Timor-Leste. Despite resource restrictions discussed by participants, interview data and social network analysis revealed evidence of information and resource sharing among organisations working within the health and social (disability and violence support) sectors in Timor-Leste (network density = 0.55 and 0.30 for information and resource sharing, respectively). Contrary to the assumption that mental health services and system strengthening are led by the Ministry of Health, the mixed-methods data sources identified a split in stewardship for mental health between subnetworks in the health and social sectors (network degree centralisation = 0.28 and 0.47 for information and resource sharing, respectively).

**Conclusions:**

Overall, the findings suggest that there may be opportunities for intersectoral collaborations in mental health systems in LMICs which do not exist in settings with more formalised mental health systems such as HICs. Holistic understandings of health and wellbeing, and a commitment to working together in the face of resource restrictions suggest that intersectoral collaboration can be employed to achieve people-centred mental health care in Timor-Leste.

## Background

People-centred approaches to mental health care are increasingly promoted in low- and middle-income countries (LMICs) through global mental health policy, practice and research directives [1, 2]. The World Health Organisation defines people-centred health care as: “*an approach to care that consciously adopts the perspectives of individuals, families and communities, and sees them as participants as well as beneficiaries of trusted health systems that respond to their needs and preferences in humane and holistic ways*.” [3]. People-centred health care is proposed to apply to people with all types of health conditions.

Intersectoral collaboration is one of the key strategies for achieving people-centred health care in the World Health Organisation Framework on Integrated People-Centred Health Services (WHO IPCHS) [3]. There is no definitional consensus on intersectoral collaboration. In line with recent conceptual developments in global health, we adopt a broad definition of intersectoral collaboration for mental health as: any planning, information and resource sharing to institute mental health care between organisations from different sectors (i.e. public, private, not-for-profit) and/or across thematic areas (i.e. health, social services) [4, 5]. This definition encompasses collaborations for mental health service referrals and back referrals, as well as for the purposes of mental health system governance, including the involvement of mental health service user and family organisations.

Emerging from the 1978 Declaration of Alma Ata [6], and subsequent action to embed Health in All Policies (HiAP) [7, 8], intersectoral collaboration underpins current global movements to achieve health equity and sustainable development [9]. Intersectoral collaboration is fundamental to the provision of people-centred mental health care because many of the sociocultural and economic determinants of mental health and wellbeing lie outside the health sector [10–12]. Furthermore, in many LMICs, people rely on customary (traditional, religious or faith-based) or private mental health providers, particularly in the absence of well-developed public health infrastructure [13–15].

Intersectoral collaboration for mental health has been shown to be effective. A systematic review of research from high-income countries (HICs) revealed that collaboration between mental health and non-clinical services improves clinical recovery and other outcomes for mental health service users (e.g. employment, housing stability), as well as system outcomes (e.g. service and cost efficiency) [16]. Such collaborations included service co-location, joint interorganizational training and use of a shared information system between services [16].

However, intersectoral collaboration is difficult to achieve. Collaboration is often challenged by systemic factors (e.g. inadequate resourcing, lack of shared interorganisational structures, goals, and trust) and interpersonal factors (e.g. poor communication) [5, 17–20]. In many LMICs, partnerships are challenged because Ministries of Health are hierarchically structured and seen as solely responsible for health activities [19]. Hence, there may be feasibility issues for promoting intersectoral collaboration for mental health in LMICs.

Despite the global imperative to increase the people-centredness of mental health care in all countries [2, 3], there is a dearth of research investigating intersectoral collaboration for mental health care across the multitude of sociocultural and resource settings that constitute the grouping LMICs. To fill this knowledge gap, this study was conducted in Timor-Leste, a LMIC in South-East Asia in the process of strengthening its public mental health system.

### Study setting: Timor-Leste

Timor-Leste is a small island nation of 1.3 million people [21]. Promoting mental wellbeing is a government priority in Timor-Leste due to a range of sociocultural and economic risk factors for distress including poverty, unemployment, and past and continuing experiences of violence [22, 23]. Rigorous estimates of the population prevalence of mental illness are limited and inconsistent. The only household survey of mental illness in Timor-Leste was conducted in 2004 with 1544 adults in the aftermath of the conflict, and estimated an adjusted 5.08% population prevalence of mental disorders [24]. However, this estimate is now 15 years old and likely does not represent the burden of mental illness in present day, more stable Timor-Leste. As well, their validity is weakened by the predominantly urban sample and the use of assessment tools that may have missed culturally meaningful idioms of mental distress. The 2016 Global Burden of Disease study estimates a 11.6% prevalence of mental and substance use problems [25].

Multiple stakeholders are involved in mental health care in Timor-Leste. Family and civil society including customary healers are the main form of support for Timorese people with mental health problems [26, 27]. Within government, responsibility for mental health is split between the Ministry of Health (MoH) and the Ministry of Social Solidarity and Inclusion (MSSI). MoH coordinates the integration of a basic package of mental health care into primary health care, and the training and deployment of the mental health workforce [28]. Community-based mental health care is mainly provided by mental health nurses, and there is one psychiatrist and one psychologist working in the National Hospital. MSSI coordinates the 2012 National Disability Policy [29], and the social protection program and disability pension, which some people with psychosocial disability resulting from mental illness receive. Ministries of Education and Justice are involved peripherally with the institution of education and legal systems that some people with mental illness have contact with. NGOs provide a psychosocial rehabilitation service (Pradet), long-term stay service (Klibur Domin) and inpatient psychiatric service (São João de Deus, Laclubar). Social and violence support NGO services including for victims of family violence and legal assistance are also accessed by some people with mental health problems. International development organisations provide financial and in-kind support to MoH, MSSI and NGO service providers through health, and disability- and gender-inclusive development activities [30].

Intersectoral collaboration is a key strategy of the yet-to-be implemented Timor-Leste National Mental Health Strategy 2018–2022, which aims to provide “*comprehensive culturally*-*appropriate community*-*based mental health and social services*” [22]. To achieve this, the National Strategy specifies collaborations between mental health, general health, maternal and child health and social support services.

However, it is not known how prevailing collaboration is structured and operates between the different stakeholders involved in mental health care in Timor-Leste. This is important to understand given the limited human and financial resources for mental health in Timor-Leste, which have been identified as barriers to collaboration in other settings. Specifically, there are only three mental health professionals per 100,000 people, and less than 0.29% of the 2018 government budget was allocated to the Public Health Directorate (including mental health) [31].

Hence, this study aimed to investigate intersectoral collaboration for people-centred mental health care in Timor-Leste’s mental health system. The study aimed to answer the following research questions:

1. To what extent is intersectoral collaboration for mental health outlined in existing government, NGO, civil society and international agency documents in Timor-Leste?
2. What are the perspectives and experiences of multiple stakeholders about intersectoral collaboration for mental health?
3. What is the strength and structure of intersectoral collaboration in the national mental health system?

This research builds upon previous research by the authors that informed the Timor-Leste National Mental Health Strategy [27], and was conducted to inform the implementation of this Strategy.

## Methods

### Study sites

Dili, the capital of Timor-Leste, was selected as a research site to understand intersectoral collaboration across national government ministries, the national hospital, NGOs (including Pradet and Klibur Domin), and international organisations. Baucau municipality in Eastern Timor-Leste, and its administrative post, Venilale, provided a comparison of collaborative processes at sub-national levels. Baucau municipality is host to the country’s second largest city where there are sub-national government ministry offices, a municipality referral hospital providing mental health care, and mental health and social support NGO service providers [32]. Venilale is a mountainous rural township which has an administration office and a government health clinic providing outreach mental health care to the surrounding villages. Laclubar administrative post in Manatuto municipality was also included as a data collection site because it hosts the São João de Deus inpatient mental health facility.

### Design

This research employed a mixed-methods convergent design to investigate intersectoral collaboration for people-centred mental health care in Timor-Leste using qualitative data derived from in-depth interviews and document review, and quantitative social network analysis. The social network analysis findings enhanced understandings derived from document review and interview data to provide a holistic and rigorous picture of intersectoral collaboration that would not have been possible using only the qualitative data [33]. This article reports findings from the third component of a larger study investigating people-centred mental health care in Timor-Leste [34].

#### Document review

A review of electronic documents was conducted to provide information about the policy context, plans and implementation of intersectoral collaboration for mental health care in Timor-Leste (research question 1). Documents reviewed were produced between 2002 and 2019 by government, NGO, civil society and international organisations, including strategic plans, policies, legislation, and reports (*n *= 33). Key documents were sourced by conducting internet or reference list searches between September 2017 and March 2019 or were provided by participants during data collection. Information emerging from the document review was interrogated further during interviews, and compared against interview data during analysis.

#### Semi-structured interviews

In-depth semi-structured interviews were conducted to ascertain the experiences and opinions of multiple stakeholders about intersectoral collaboration for mental health (research question 2). Interviews were conducted with 85 adults (≥ 18 years) who were: (1) mental health service users (*n* = 20) and their families (*n* = 10); (2) government decision makers (*n* = 10); (3) mental health and social service providers (*n* = 23); (4) civil society (*n* = 9); and (5) other groups including international development organisations involved in mental health or social policy or service delivery (*n* = 13, see Table 1). Mental health service users were defined as adults aged 18 years or older who had used health or social support services related to their mental health and were able to provide informed consent and respond to interview questions. In the absence of a Timorese culturally-validated psychiatric diagnostic tool, the definition of mental illness was intentionally kept broad to capture the range of people who were considered to use services for mental illness. Mental health service users and their families were recruited through the administrative post health staff in Venilale and NGO service providers in Dili. Participants in groups 2 to 5 were recruited purposively by First Author TH based on their positions in government, NGO, international development and civil society organisations and institutions. In the first instance, participants were identified through a document review and the existing research collaborations that supported the development of the National Mental Health Strategy. Snowball sampling was used to identify and recruit subsequent participants who were mentioned in interviews and not already identified. Data were collected from September 2017 to August 2018.

**Table 1:** Participant demographics. Table adapted from [62]

	Mental health service users		Family members		Service providers		Decision makers		Civil society		Other community members and organisations		Total	
N	20		10		23		10		9		13		85	
	**n**	**%**	**n**	**%**	**n**	**%**	**n**	**%**	**n**	**%**	**n**	**%**	**N**	**%**
Age														
26–40	12	60	2	20	10	43.5	1	10	4	44.4	6	46.2	35	41.2
41–55	6	30	5	50	8	34.8	8	80	3	33.3	5	38.5	35	41.2
56–70	2	10	3	30	5	21.7	1	10	2	22.2	2	15.4	15	17.6
Gender														
Male	7	35	7	70	13	56.5	9	90	8	88.9	7	53.8	51	60.0
Female	13	65	3	30	10	43.5	1	10	1	11.1	6	46.2	34	40.0
Education														
None	1	5	2	20	0	0.0	0	0	0	0.0	0	0.0	3	3.5
Primary	11	55	5	50	0	0.0	0	0	0	0.0	0	0.0	16	18.8
Secondary	4	20	1	10	1	4.3	0	0	4	44.4	3	23.1	13	15.3
Tertiary	4	20	2	20	22	95.7	10	100	5	55.6	10	76.9	53	62.4
Location														
Dili	5	25	0	0	15	65.2	5	50	6	66.7	9	69.2	40	47.1
Baucau	2	10	1	10	4	17.4	4	40	0	0.0	3	23.1	14	16.5
Venilale	13	65	9	90	3	13.0	1	10	3	33.3	1	7.7	30	35.3
Laclubar	0	0	0	0	1	4.3	0	0	0	0.0	0	0.0	1	1.2

We adopt WHO’s definition of civil society as individuals and organisations working for “*collective action around shared interests, purposes and values, generally distinct from government and commercial for*-*profit actors*” [65]. Civil society includes community groups, social movements and advocacy groups. Civil society also includes local chiefs and customary healers who may not be mobilised in formal groups. Other community members and organisations include representatives from international development agencies, law enforcement, universities, and other people with relevant knowledge but who do not work specifically in mental health in Timor-Leste

Interviews were semi-structured using an interview guide tailored to participant type. The interview guide was structured around the five strategies of the WHO Framework on Integrated People-Centred Health Services (2016): engage service users; strengthen governance; re-orient the model of care; forge intersectoral collaboration; and foster an enabling environment. This article reports findings pertaining to intersectoral collaboration. The interview guide contained open-ended questions and quantitative measures of collaboration. Open-ended interview questions enquired about the experiences, structures and processes of mental health service delivery and policy making (see Interview guides in Additional file 1). Quantitative measures are outlined below in “Descriptive social network analysis”. The interview guides were translated, their meaning checked, and piloted before data collection commenced. Author TH conducted all interviews directly in English, or with a trained interpreter in Tetum or Portuguese (national languages) or several Baucau local languages (Makassai and Cairui). Interviews lasted on average 47 min (range 7 to 111 min), and were in private places, including workplaces, health facilities or community houses.

Framework analysis, an inductive and deductive qualitative data analysis method [35], was used to analyse interview data in NVivo version 12 [36]. Author TH conducted the framework analysis and an independent researcher validated coding. Author TH employed a combination of emergent themes and a priori codes (e.g. enabling factors, barriers). This article reports three main themes and 15 sub-themes relevant to intersectoral collaboration. Preliminary results were presented back to participants and interested parties in communities in Dili and Venilale to verify the authors’ interpretation of the data.

#### Descriptive social network analysis

Intersectoral collaboration, as well as being difficult to achieve, is difficult to measure with traditional methods. Intersectoral collaboration can be considered a type of networked relationship [17]. Social network analysis (SNA), a complex systems discipline and quantitative methodology, is widely used in HICs to measure health policy networks [37–40]. SNA has more recently been applied in LMICs [41–45] in line with calls to adopt systems thinking to understand health system governance in these contexts [19]. For example, Hagaman et al. demonstrated the utility of SNA for understanding surveillance systems for suicide in Nepal [45]. Prior to our study, SNA had not been used to investigate both mental health service and system governance networks in a LMIC.

We used SNA to measure the strength and structure of connections between organisations operating at the national level of the mental health system in Timor-Leste (research question 3). SNA complemented the understanding about intersectoral collaboration garnered through qualitative data by examining the role of each organisation in the mental health network, as well as properties of the overall network [46].

SNA methods are summarised in Table 2. For SNA, the network was defined as 27 organisations from government, NGO, civil society and other organisations working in national mental health and social care (participant categories 2 to 5). Organisations were identified through previous research informing the National Mental Health Strategy 2018–2022 [27] and the document review. There were insufficient numbers of mental health organisations at sub-national levels to conduct SNA. As stated above, stakeholders were recruited using purposive and snowball sampling methods because SNA seeks to understand collaborative patterns between specific stakeholders and randomisation is unlikely to incorporate all central stakeholders [47].

**Table 2:** Stages of social network analysis. Table adapted from [50]

Stage	Processes and measures
1. Defined the network	i. Listed all organisations involved in the national mental health system based on previous research and document review ii. Supplemented list with additional organisations identified through snowballing during interviews
2. Defined the relationships between organisations	iii. Displayed the list of organisations in a table iv. During interviews, asked participants with knowledge of their organisation about the relationship between their organisation and other organisations v. Two quantitative indicators were collected. Participants rated the frequency of contact and frequency of resource sharing over the preceding year vi. Once all responses were received, scores from each organisation were combined into a single matrix for each key indicator
3. Analysed the structure of the system using UCINET to generate measures	Network metrics i. Density ii. Average degree iii. Average distance Organisation metrics i. In-degree centrality ii. Betweenness

SNA questions were embedded in interviews with one participant from each national organisation with knowledge of operations (i.e. manager level). These participants were presented with a list of organisations and asked about connections between their organisation and these listed organisations. These participants also nominated any missing organisations that they worked with. This ‘recall list’ is a validated technique for prompting participants to accurately report connections [48].

Two widely-used quantitative SNA indicators were collected. Participants rated the frequency of contact/information sharing (e.g. meetings, phone calls, emails) and the frequency of resource sharing (e.g. funding, building space, transport, printing, materials) between their organisation and others over the preceding year on a six-point scale (*none*, *yearly*, *quarterly*, *monthly*, *weekly*, *daily*). Resource sharing is assumed to indicate a stronger degree of relationship than information sharing [5]. If there was overlap in categories (e.g. car sharing to transport patients involved both contact and resource sharing), participants rated contact and resource sharing separately.

Descriptive quantitative analysis of the two SNA indicators was conducted using UCINET software [49]. SNA data resulted in one matrix for demand and a second matrix for supply of information/resource sharing [50]. The rows in each matrix corresponded to the 27 organisations and were inputted with the frequency rating for information/resource sharing such that 0 indicated no relationship and 1–5 indicated an ascending order of connection. For each indicator, a network dataset was produced by combining these demand and supply matrices into a single matrix [48]. UCINET mapped each network and generated network-level and organisation-level metrics [49] (see Table 3 for a definition of each metric). Data cleaning was conducted in Microsoft Excel. Missing values for three organisations who were not interviewed were replaced with connection ratings reported by organisations who did respond [51].

**Table 3:** Definition of key network and organisation metrics. Table contents adapted from [47]

Metric	Definition and mental health system interpretation
Network metrics	
Density	Ratio of the number of connections to the number of possible connections in the network. A dense network indicates that organisations are well-connected and information/resources flow rapidly between them
Average degree	Average number of relationships in the network. Like density, this assumes that more connections indicate greater information/resource flow between organisations
Average distance	Number of connections that separate two organisations, whereby an average distance of 1 indicates that all organisations are directly connected
Degree centralisation	Ratio of the sum of the differences in centrality between the most central organisation and all other organisations in the network to the largest possible sum of these differences. Higher values indicate a more centralised network
Organisation metrics	
In-degree centrality	Number of direct connections an organisation has with other organisations as reported by partnering organisations. A measure of the importance of each organisation. Identifies which organisations act as stewards organisations in the network
Betweenness	Extent that an organisation is located on the path between other organisations (indirect connections). The extent that an organisation is a bridge between other organisations

### Ethics

Verbal or written consent (depending on participant preference and literacy) was provided before interviews commenced and were audio recorded. Participants responding to SNA questions provided separate consent to include their organisation. Participant quotations and organisations in SNA were de-identified to fulfill the governing ethics agreements. Ethical approval was granted by University of Melbourne Human Ethics Sub-Committee (HESC: 1749926) and National Institute of Health in Timor-Leste (1070MS-INS/DE-DP/CDC-DEP/IX/2017).

## Results

The results section presents a synthesis of qualitative findings from the document review and interviews, and separately reports social network analysis findings. The mixed-methods findings are integrated in “Discussion”. Table 4 presents the framework analysis themes and sub-themes for intersectoral collaboration from interviews and documents (research questions 1 and 2). See Additional file 2 for a summary table of extant government strategy, policy and legal documents related to mental health and psychosocial disability in Timor-Leste (research question 1).

**Table 4:** Framework analysis themes and sub-themes for intersectoral collaboration

Theme	Sub-themes
1.1 Enabling factors	Importance of intersectoral collaboration
	Responsibility of all
	Address broader determinants of mental health
	Different roles for health and social sectors
1.2 Barriers	Social importance of mental health
	Resource restrictions
	Competing demands on government
1.3 Intersectoral collaboration for policy making and planning	Ministerial working groups
	Social sector working groups
1.4 Intersectoral collaboration for service delivery	Customary healers
	Government health providers
	NGO service providers
	Authorities
	Social sector
	Disability
	Violence support organisations

### Interviews and documents: perspectives and experiences about and documented approaches to intersectoral collaboration

#### Enabling factors for intersectoral collaboration

The importance of intersectoral collaboration for mental health was a prominent theme across participant interviews and documents. Intersectoral collaboration between ministries, public institutions, development partners, civil society and communities was a key strategy in the National Mental Health Strategy of Timor-Leste 2018–2022 [22], National Disability Policy 2012 [29], and Disability Action Plan (unapproved) [52]. One MoH representative advocated for: “*socialis[ing] all the other institutions and NGOs so they know that they can’t only walk their part, [mental health is] not only [the responsibility of] Ministry of Health.*” (Decision maker #5, 36–40 years, male). A Baucau service provider explained that intersectoral collaboration was important because of the broader drivers of mental health:

*Mental health is not only the responsibility of the health [sector]. For example, people have problems with food, with money, so we all need to work together to collaborate to provide treatment for people with mental health problems. The community, the families and the local authorities need to work together.* (Service provider #4, 46–50 years, male)

Similarly, a MSSI representative described complementary roles for MoH and MSSI in mental health, such that MSSI provided food and MoH provided medication for families affected by mental ill-health: “*because [people with mental illness] need to eat in order to take medication*” (Decision maker #9, 46–50 years, male).

#### Barriers to intersectoral collaboration

Despite the emphasis on intersectoral collaboration, mental health had limited specific mention in key health, social sector and development strategies (e.g. National Health Sector Strategic Plan 2011–2030, and Strategic Development Plan 2011–2030) [53, 54]. One civil society representative said the lower priority of mental health reflected social norms: “[*mental health] is not socially talked about, or socially an important subject, so people are not really looking at it as something that they need to focus on*” (Civil society #6, 26–30 years, male).

Government and civil society participants identified a lack of resources as a challenge to government services working with the NGO sector: “*So far only Pradet [NGO] have good knowledge and experience with these people [with mental illness] because the government have very limited resources”* (Civil society #5, 36–40, male). A development partner explained that the mental health-relevant portfolios within MoH and MSSI received less political and fiscal priority:

*Mental health is so poorly funded under [MoH] and those people are not very powerful within the [MoH], and likewise people who work in disability within [MSSI] are not very powerful within the ministry and have very low funding as well* (Other #1, 36–40 years, female)

Government decision makers and community members stated that the demands on government to address Timor-Leste’s other economic, political and social development challenges meant that ministries who were not directly responsible for mental health did not prioritise working intersectorally in this area:

*There are a lot of issues in Timor, not only mental health. [The government] also try to solve malnutrition, and improve access to clean water, education, a lot of things.* (Other #4, 30–35 years, female)

#### Intersectoral collaboration for policy making and planning

Participants and documents reported many links between health and other sectors in Timor-Leste. Decision makers and documents reported that there were national- and municipality-level ministerial working groups for health and disability programming between MoH, MSSI and Ministry of Education. Government and NGO service providers said they attended quarterly disability or social sector working group meetings at the national and municipality levels. One decision maker from Baucau explained:

*In Baucau, we have a working group to deal with cases of [people requiring] psychosocial recovery that is composed of the Ministry of Health, the Ministry of Social Solidarity, Pradet [NGO], Alfela [NGO], Ministry of Public Administration, and civil society like safe houses [for female and child victims of violence]. We have a quarterly meeting so we discuss all the things related to these cases. Every institution comes together and presents the issues they are facing and discusses their priorities and actions.* (Decision maker #3, 46–50 years, male)

There is no mental health service user or family organisation in Timor-Leste so participants did not report contact with service users and families as a key part of their collaborations with other organisations.

#### Intersectoral collaboration for service delivery

**Fig. 1:**
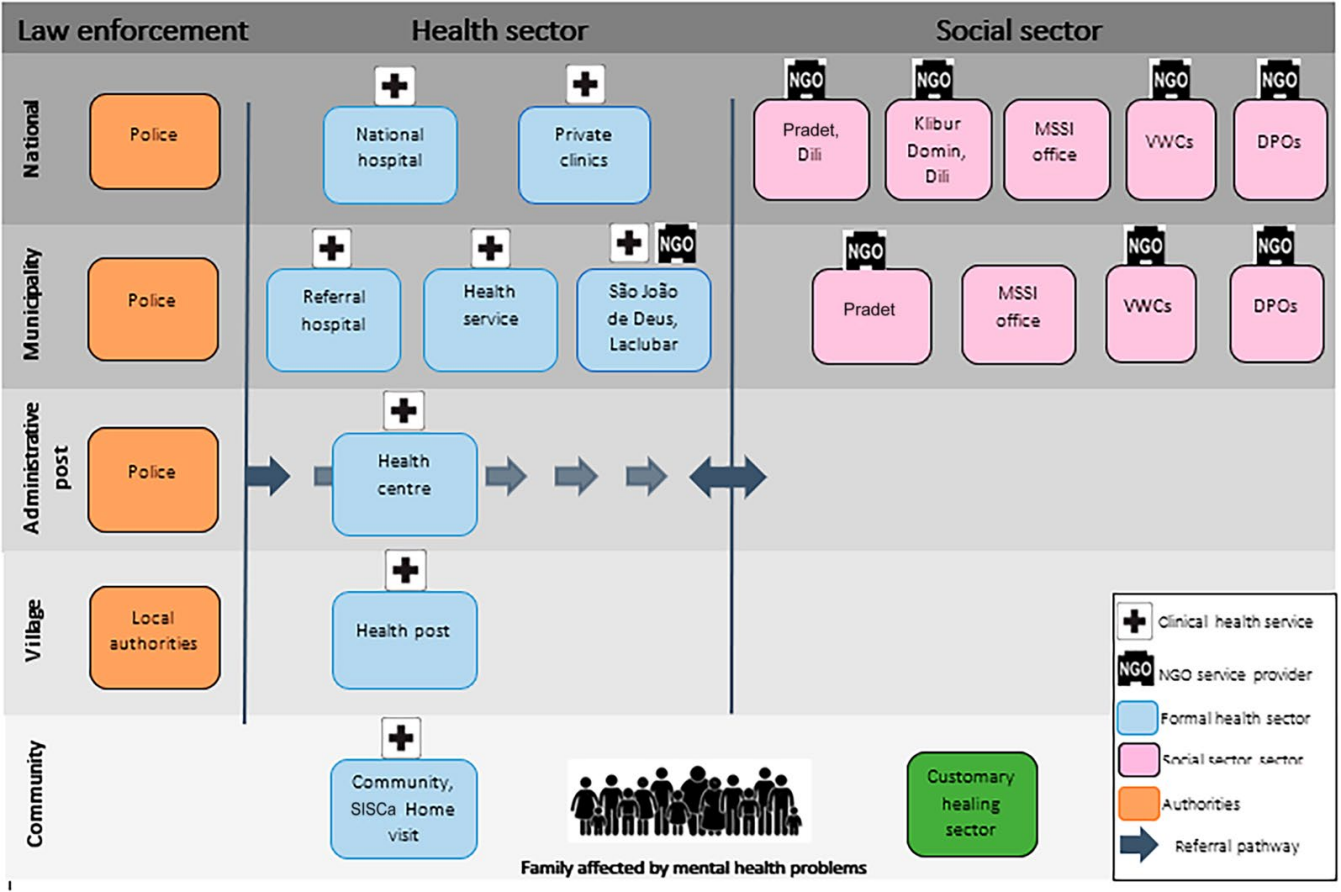
Mental health and social service referral and back referral pathways across multiple levels of the mental health system. *MSSI* Ministry of Social Solidarity, *VWCs* violence, women and children organisations, *DP0s* Disabled Persons Organisations, *SISCa* Integrated Health Services, Outreach Care

Figure 1 displays the key stakeholders for mental health and social service delivery across multiple levels of the mental health system based on information reported in interviews and documents. Participants reported that families affected by mental health problems directly accessed support from customary healers, government health services, Pradet or private health clinics. Police, local authorities, private clinics, social sector providers and customary healers referred people with mental health problems to government health facilities and Pradet. Referrals were made to and from government health services and Pradet, and São João de Deus inpatient mental health facility if the person was deemed to be very unwell. Government health services and Pradet also referred to, and received referrals from, MSSI and disability, violence or women’s support organisations. Klibur Domin, a disability NGO, provided a longer stay service for people with mental illness coming to/from: family, São João de Deus mental health facility, prison or from living in homelessness. This quotation from a service provider exemplifies the information provided by participants:

*We have a network with other organisations, they are our partners. These organisations are all over Timor*-*Leste, from Dili to Viqueque [municipality], to Lospalos [municipality], Suai [municipality], Maliana [municipality]. We have good communication and coordination with these partners so that we can give assistance to the clients from wherever they are from [in Timor*-*Leste].* (Service provider #3, 36–40 years, female)

### Descriptive social network analysis: the strength and structure of national-level intersectoral collaboration

#### Network metrics

Network metrics are provided in Table 5. The contact network had greater connectivity than the resource network, as indicated by higher density and average degree scores. Approximately 50% of organisations reported directly sharing information compared to 30% who directly shared resources (density = 0.55 and 0.30 for contact and resource sharing, respectively).

**Table 5:** Network metrics for the contact and resource sharing networks of the national mental health system

Network metric	Contact network	Resource sharing network
Density	0.55	0.30
Average degree	14.22	7.70
Average distance	1.50	1.80
Degree centralisation	0.28	0.47

See Table 3 for a definition of each metric

**Fig. 2:**
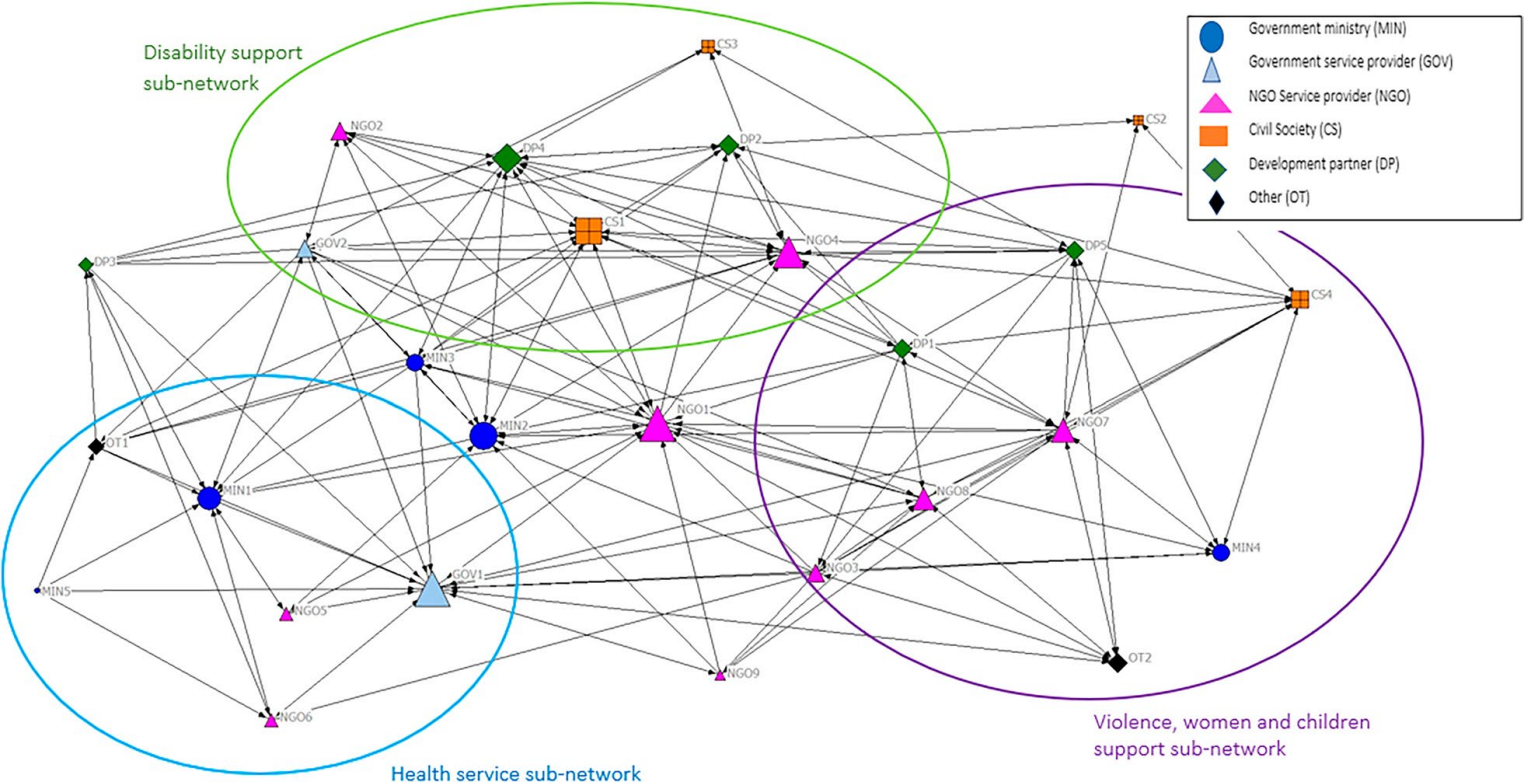
Displays a map of the intersectoral connections between 27 organisations working in the national level of the mental health system based on frequency of contact (information sharing) over the preceding year. The lines connecting organisations in each map represent connections at least once a month (i.e. monthly, weekly, daily)

**Fig. 3:**
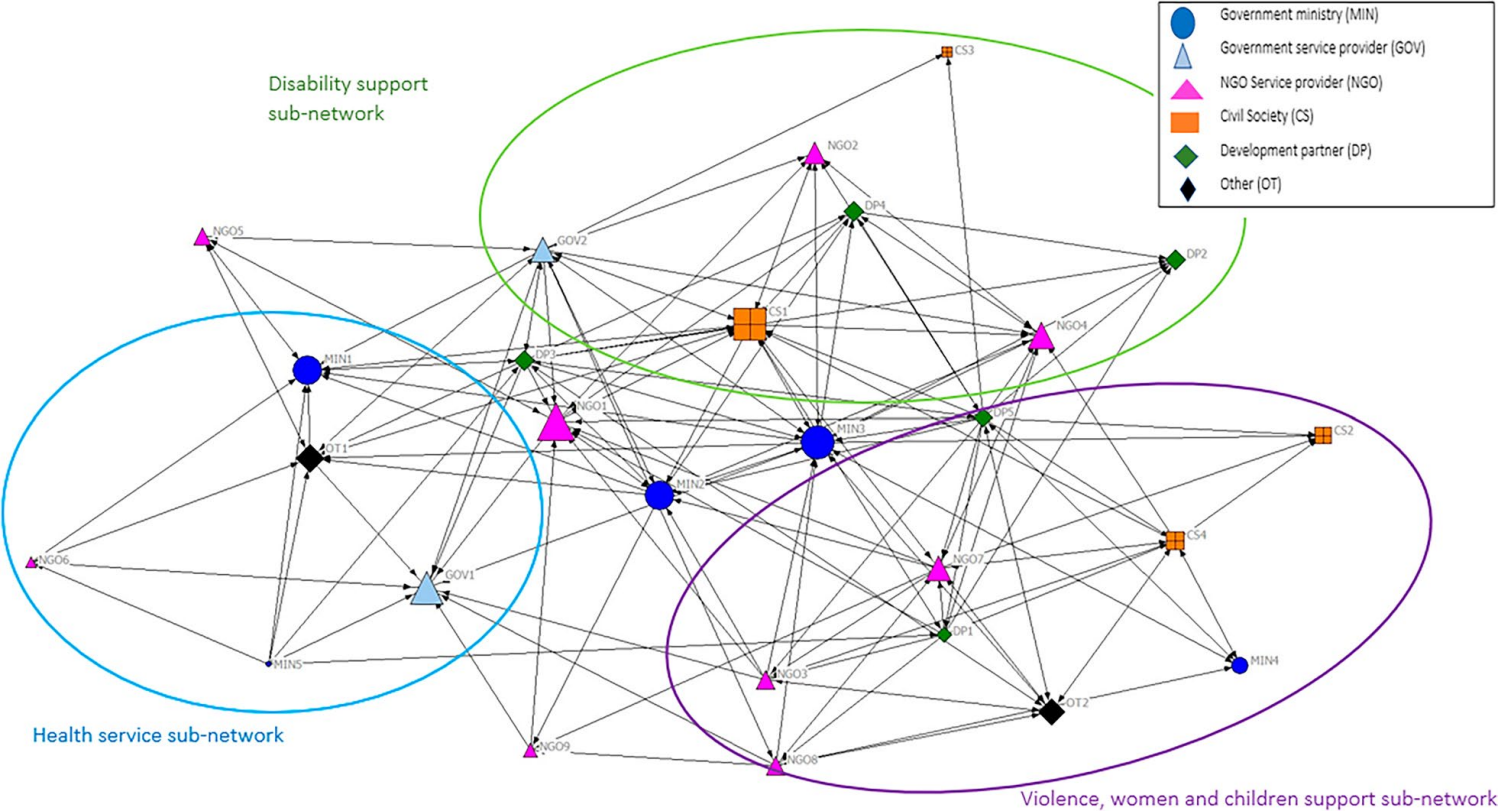
Shows the intersectoral connections between these organisations based on the frequency of resource sharing at least monthly

More organisations had direct contact for information sharing than resource sharing (average distance estimates = 14.22 and 7.70, respectively). As indicated by Figs. 2 and 3, the networks for information and resource sharing were similarly distributed indicating that the same organisations (e.g. NGO1, MIN2, MIN3, CS1) played a central role in both types of collaboration. Three sub-networks emerged for both information and resource sharing within the national mental health system: (1) health, (2) disability, and (3) violence, women and children’s support. As indicated in the key on Figs. 2 and 3, these subnetworks constituted different types of organisations, including government ministries, NGO and government service providers, civil society, etc. These sub-networks are displayed as rings in Figs. 2 and 3 and corresponded to the governance structures described by participants and documents, which split mental health between the health and social sectors. These sub-networks indicated that the mental health network was relatively decentralised, as indicated by network degree centralisation estimates of 0.38 and 0.47 for information and resource sharing, respectively.

#### Organisation metrics

Metrics were calculated to identify the relative importance of organisations in terms of their number of direct connections (in-degree centrality) and indirect connections (betweenness). Organisations with more direct or indirect relationships are assumed to have more opportunities to access relevant information or resources [42]. One NGO service provider (NGO1) and three government organisations (GOV1, MIN1 and MIN2) had the most direct and indirect connections for information sharing, and direct connections for resource sharing. International development organisations and civil society stakeholders (OT1, DP5 and CS1) had the most indirect relationships for resource sharing.

## Discussion

This study is the first to investigate intersectoral collaboration for both mental health service provision and mental health system governance in a LMIC using mixed-qualitative methods and social network analysis (SNA). The key findings were:

1. Consensus among stakeholder groups that intersectoral collaboration for mental health is important in Timor-Leste;
2. Information and resource sharing exist among organisations (e.g. government, NGO, civil society, international development) working within the health and social (disability and violence support) sectors, despite resource restrictions discussed by participants; and
3. SNA proved useful for identifying subnetworks of intersectoral organisations to substantiate data from interviews and documents such that there was a split in stewardship for mental health between subnetworks in the health and social sectors.

The functional intersectoral connections within the Timor-Leste mental health system contrast with the challenges of health governance reported in other LMICs (e.g. weak government institutions, hierarchical structure of MoH) [19]. Intersectoral collaboration for mental health in Timor-Leste may be facilitated for several reasons. First, the appreciation of the interconnections between mental health and other sectors displayed by Timorese participants reflected the holistic understandings of health found in Timor-Leste [55] and indigenous peoples around the world [56, 57]. Second, connections across the mental health system may have been enabled because they were primarily forged to share information, which is assumed in social network science to indicate a less intensive type of collaboration than resource sharing [5]. However, given that health knowledge is often among the most valuable of resources in LMICs [58], this finding could also suggest a stronger degree of collaboration. Third, connections between organisations may be forged out of necessity given the low availability of human and financial resources for mental health in Timor-Leste. Fourth, the relatively small number of organisations working in mental health and social services in Timor-Leste (*n *= 27) created a bounded community of practice, which contrasted with the fragmentation of mental health and social service systems reported to challenge collaboration in HICs [16]. The tightly-defined network combined with the reliance on informal and kinship networks for health previously reported in Timor-Leste [59] may overcome barriers to trust reported in settings with more formalised systems of mental health governance [17, 18]. This is also in line with broader governance literature which reports that collaborations are most effective when they have clearly defined and agreed upon understandings of which problems they will address [60]. Hence, it will be important to consider how to maintain these connections as the Timorese mental health system expands and formalises; a key concern for mental health system strengthening in other LMICs.

Despite these information and resource sharing collaborations, the document review highlighted that mental health had limited specific mention in other key government policies. The commitment to intersectoral collaboration expressed by our participants may not be shared by other stakeholders who are not currently engaged with the mental health system. Thus, the disadvantage of not integrating mental health into intersectoral policies is that resources and political will cannot be mobilized to translate intention into practice [8]. Timor-Leste could benefit from explicitly incorporating mental health into intersectoral policies in line with efforts throughout the Asia and Pacific region to place ‘Health in All Policies’ (HiAP) [8, 61]. Increasing awareness and understanding of the importance of mental health among intersectoral stakeholders may be part of achieving this. Given the overlap in scope, people-centred mental health care as a concept would benefit from more explicitly aligning with existing global health movements for universal health coverage and HiAP to relish the learnings and progress already made in these areas over the past 40 years.

The shared stewardship for mental health in Timor-Leste is contrary to the assumption that the health sector is the primary steward for the people-centred health care model. This split stewardship is beneficial in Timor-Leste because it allows for more efficient use of existing resources and also opens up funding channels for mental health service providers through disability- and gender-inclusive development that are not available through traditional health financing [30]. The central role of the social sector in the mental health system may promote people-centredness because social sector activities tackled the social exclusion of people with mental health problems and their families in Timor-Leste found in previous research (e.g. experiences of stigma, exclusion from employment and education) [62], which are also key barriers to mental health care access [63]. This governance structure acknowledges the social determinants of mental health and the co-existing health and social issues affecting families, which are typically under-addressed when there is a myopic focus on treating the mental illness. On the other hand, as one participant explained, government focus on mental health may be diluted without one central champion [19]. Furthermore, if more resources flow into mental health in Timor-Leste, requiring a greater level of coordination than information sharing, parallel systems of care may emerge over time. Hence, a key consideration is how to ensure that there are no gaps in implementation of strategies to achieve people-centred mental health care in Timor-Leste and other LMICs in which mental health stewardship is shared. This finding also highlights that global mental health efforts should not presume that that Ministry of Health is always the primary steward of mental health.

The prevailing collaborative structures for mental health service delivery and governance in Timor-Leste have important implications for the implementation of Timor-Leste National Mental Health Strategy 2018–2022. Currently, the key role of the social sector in mental health governance is underestimated. Decisions need to be made as to whether the split stewardship for mental health continues or if MoH steps up to lead mental health initiatives in line with their mandate established in the National Strategy. The service delivery collaborations highlighted the importance of social sector NGO service providers (e.g. psychosocial rehabilitation, violence support services), which suggests that training and capacity building that is currently focused on government mental health service providers should also incorporate these NGO providers. Finally, the absence of a mental health service user and family organisation is a key consideration for people-centred mental health care in Timor-Leste because without such a mechanism, the involvement of mental health service users and families in future intersectoral collaborations will likely remain minimal [64].

Our study had several limitations. SNA data may not have accurately captured the dynamic nature of relationships between organisations because it was cross sectional; assumed that information and resource sharing indicated relationship quality; and relied on participants accurately reporting connections with other organisations. However, we are confident that SNA accurately measured and mapped collaboration because SNA findings triangulated with data from interviews and documents. Our study is also limited because we did not incorporate the role of the customary sector, who we know from previous research by the authors plays a large role in the provision of mental health care in Timor-Leste and have emergent collaboration with the formal mental health sector [27]. Future research could use SNA to examine collaborations between the formal mental health and customary sectors over time. Research could also investigate the informal processes that drive intersectoral collaboration in Timor-Leste (e.g. trust) so that these can be harnessed to develop the mental health system.

## Conclusion

Overall, the findings suggest that there may be opportunities for intersectoral collaborations in mental health systems in LMICs. These may not exist in settings with more formalised mental health systems such as HICs in which systemic (e.g. service fragmentation) and interpersonal factors (e.g. poor communication) are barriers to working collaboratively. The holistic understanding of health and wellbeing, and the commitment to working together in the face of resource restrictions suggest that intersectoral collaboration can be employed to achieve people-centred mental health care in Timor-Leste. Intersectoral collaboration is not a new idea, and the people-centred mental health care model may have more uptake if it is tied to existing movements to reduce health inequities and ensure sustainable development.

## Supplementary information

**Additional file 1.** Interview guides.

**Additional file 2: Table S1.** Summary of key government strategies and law pertaining to mental health in Timor-Leste.

## Data Availability

Participants shared their opinions and experiences upon assurance that their confidentiality and anonymity would be protected. Hence, the research data is not available publicly because this would compromise individual privacy and our ethical approval conditions.

## Abbreviations

DPOs: Disabled Persons Organisations
HiAP: Health in All Policies
SISCa: Integrated Health Services Program
HIC: high-income country
LMIC: low- and middle-income country
MoH: Ministry of Health
MSSI: Ministry of Social Solidarity and Inclusion
NGO: Non-Government Organisation
SNA: social network analysis
VWCs: violence, women and children organisations
WHO: World Health Organisation
WHO IPCHS: WHO Framework on Integrated People-Centred Health Services
