# Supplementary material for: Intersectoral collaboration for people-centred mental health care in Timor-Leste: a mixed-methods study using qualitative and social network analysis
Source: Int J Ment Health Syst. 2019 Nov 16;13:72. doi: 10.1186/s13033-019-0328-1 (PMC6858633; doi:10.1186/s13033-019-0328-1)
Supplement: Supplementary file 1 — Additional file 1. Interview guides. [file 13033_2019_328_MOESM1_ESM.docx]

# Interview guides

## Guide 1: Consumer/family member interview guide

**Introduction**

Hello my name is Teresa Hall and I am a student from the University of Melbourne in Australia conducting a study on mental health care. I am here today to talk with you about mental health care in Timor-Leste. I am interested in the aspects of mental health care that are working well, and those that are not working well, based on your own opinions and experiences. The aim of this study is to contribute to improvements in the mental health system. You have agreed to take part in the interview and for me to audio record it. It will take about one hour. Please let me know if you need a rest or want to stop the interview. Remember:

- Please tell me honestly what you think about mental health care.
- I will ask questions for you to answer as you please.
- There are no right or wrong answers. Your real stories and thoughts are important.
- I won’t discuss what you tell me with anyone else; it is confidential.

**Mental Health**

1. Tell me about having or caring for a person who has an illness.
2. Do other people know about your illness? Who? Do they understand your illness?

**Pathway to mental health care, experience of care and service orientation**

When you first thought that you / your family member might have an illness,

1. What did you do? Did you want to get help right away or did you wait? Why?
2. Who did you ask for help when you/your family member] felt sick? Family? Clinic? Healer?
3. Who helped you? Where did they work? What support did they give you?
4. What does it take for you to stay well/ for the illness to stay away? Medicines? Talking? Rehabilitation? Any others?
5. What do you think about the health staff? Can you speak freely to them about illness & treatment? What kinds of things did they tell you about you/your family member’s illness?
6. Do you feel that you have enough understanding about your condition and its treatment? When you want to know something about mental illness, where do you generally get your information from? (friends, family, traditional healers, internet, church, organisations, service providers?)
7. Do the health staff ask you about what you or your family want, need or prefer in treatment?
   1. Is this important to you?
8. Did the health staff ask you about other things in your life? Your family? Your friends? Work?
   1. Is this important to you?
9. Do you want/need to keep seeing the mental health service? Why?
10. What would you like to be better about the mental health service?

**Engagement, empowerment and participation**

1. What gives you pleasure in your life? And what do you find most challenging?
2. Do you ever meet with other people with an illness similar to yours to talk? Do activities? Would you like to?
3. What contribution does your family make to the quality of your life? To work? To be well?
4. Does your family/do you need more help to enjoy life? What kinds of things? From who?
5. Do you know of anybody– an organisation or a person who teaches the community about mental health? What do they do?
6. Does this organisation/person help the government to make the health system better? How?
7. Would you want to be involved? Why? Why not? What would make this easy/difficult?
8. Do there need to be special services for people with a mental illness and their families? Why or why not? (day centre, clinic, community house)

**Wrap**

1. Is there anything else you want to say about mental health care in [municipality/admin post]?

Thank you very much for your participation

**Matadalan entrevista 1: ema ho moras, no sira-nia familia**

**Introdusaun**

Ha'u iha-ne'e ohin loron atu ko'alia ho Ita-Boot kona-ba tratamentu ne'ebé ema bele hetan ba moras (liuliu moras mentál nian) iha Timor-Leste. Ha'u buka informasaun kona-ba saida mak la'o di'ak, no buat ne'ebé la'o la di'ak, ka presiza atu hadi'a, tuir Ita-Boot nia hanoin, no tuir Ita-Boot nia esperiénsia. Objetivu husi peskiza ne'e mak atu hato'o informasaun ba governu no grupu seluk hodi bele halo sistema saúde mentál di'ak liu tan. Ita-Boot hatete ona ami katak Ita-Boot prontu atu foti parte iha peskiza ne'e, no prontu ba ha'u atu grava audio husi ita-nia entrevista ne'e. Entrevista ne'e sei han tempu maizumenus oras ida. Favór ida fó hatene ha'u se Ita-Boot presiza deskansa, ka hakarak para entrevista ne'e.

Informasaun adisionál kona-ba entrevista:

- Lalika ta'uk atu ko'alia buat ne'ebé tuir realidade, Ha'u hakarak rona buat ne'ebé di'ak, no mós buat ne'ebé la di'ak kona-ba sistema saúde ba moras mentál nian iha Timor-Leste.
- Ha'u sei husu pergunta, no Ita-Boot bele hatán konforme de'it Ita-Boot nia hakarak.
- Ita-Boot hatán saida de'it di'ak. Laiha resposta ne'ebé loos ka sala. Ita-Boot nia istória no hanoin mak importante.
- Ha'u nunka atu hatete ba ema seluk katak informasaun ne'ebé Ita-Boot dehan mai ha'u mai husi Ita-Boot. Bainhira peskiza ne'e hato'o rezultadu ba ema seluk, la uza ema nia naran.

**Saude mental**

1. Favór ida fó hatene ha'u kona-ba Ita-Boot nia esperiénsia moras, ka Ita-Boot nia esperiénsia tau matan ba ema ne'ebé moras.
2. Ema seluk hatene kona-ba Ita-Boot nia moras ka lae? Sira komprende kona-ba Ita-Boot nia moras ka lae?

**Tratamentu iha Timor-Leste**

Bainhira Ita-Boot foin hatene (uluk) katak Ita-Boot/ema iha Ita-Boot nia família dalaruma hetan moras ne'e:

1. Ita-Boot halo saida? Ita-Boot hakarak hetan ajuda/tratamentu kedas ka Ita-Boot hein atu hetan ajuda/tratamentu? Tanbasá?
2. Ita-Boot husu ajuda husi sé bainhira Ita-Boot/ema iha Ita-Boot nia família hetan moras ne'e? Husu família? ka husu klínika? ka husu matan-dook?
3. Sé mak ajuda ita? Sira servisu iha ne'ebé? Sira ajuda ka suporta Ita-Boot hanesan saida?
4. Ita-Boot presiza saida de'it hodi Ita-Boot nia saúde di'ak (hodi moras ne'e bele dook husi ita)? Presiza ai-moruk, ka presiza ko'alia ho ema, ka presiza reabilitasaun, ka presiza halo adat, ka presiza buat seluk?
5. Ita-Boot hanoin saida kona-ba ema ne'ebé servisu iha saúde? Ita-Boot bele ko'alia ho nakloke ho sira kona-ba Ita-Boot nia moras no tratamentu ba Ita-Boot nia moras ne'e ka lae? Sira dehan saida de'it ba Ita-Boot kona-ba ita/ema iha Ita-Boot nia família nia moras?
6. Ita-Boot sente katak Ita-Boot nia kompriensaun kona-ba Ita-Boot nia moras to'o ka lae? Ita-Boot sente katak Ita-Boot nia kompriensaun kona-ba tratamentu ba Ita-Boot nia moras to'o ka lae? Bainhira Ita-Boot hakarak hatene buat ruma kona-ba moras mentál, Ita-Boot buka Ita-Boot nia informasaun husi ne'ebé? (husi kolega, família, matan-dook, internet, igreja, organizasaun seluk? Doutór/a ka enfermeiru/a?)
7. Doutór/a no enfermeiru/a sira husu Ita-Boot kona-ba buat ne'ebé Ita-Boot hakarak ka presiza bainhira halo tratamentu ka lae?
8. “”…..buat seluk iha Ita-Boot nia moris? Ita-Boot nia família, Ita-Boot nia kolega? Servisu?
9. Ita-Boot hakarak/presiza konsulta nafatin ba Ita-Boot nia moras?
10. Tuir Ita-Boot nia hanoin, saida mak bele sai di'ak liu iha servisu saúde mentál iha Timor-Leste?

***Engagement*, Abilidade atu halo buat ruma ba Ita-Boot nia an rasik, no Partisipasaun**

1. Saida mak halo Ita-Boot sente kontente iha Ita-Boot nia moris? Saida mak dezafiu boot liu iha Ita-Boot nia moris?
2. To'o agora, Ita-Boot hasoru malu ho ema seluk ne'ebé moras hanesan Ita-Boot no ko'alia ba malu ka halo atividade ruma hamutuk? Ita-Boot hakarak hasoru malu ho ema ne'ebé moras hanesan Ita-Boot ka lae?
3. Ita-Boot nia família halo kontribuisaun saida de'it ba Ita-Boot atu halo Ita-Boot nia moris di'ak liu? Sira ajuda Ita-Boot hodi bele servisu ka lae? Sira ajuda Ita-Boot hodi bele saúde di'ak ka lae?
4. Ita-Boot ka Ita-Boot nia família presiza tan ajuda ruma atu bele enjoi imi nia moris ka lae? Presiza ajuda hanesan saida? Presiza hetan ajuda ne'e husi sé?
5. Ita-Boot koñese ema ruma ka hatene organizasaun ruma ne'ebé hanorin komunidade kona-ba saúde mentál ka lae? Sira halo saida de'it?
6. Ema ka organizasaun ne'e ajuda governu atu halo sistema saúde mentál di'ak liu ka lae? Sira ajuda hanesan saida?
7. Se Ita-Boot envolve an hodi ajuda halo sistema saúde sai di'ak liu tan, Ita-Boot hakarak envolve an hanesan saida? Saida mak halo Ita-Boot fasil liu ka susar liu atu envolve an?
8. Tuir Ita-Boot nia hanoin, presiza ka lae atu iha ajuda ka atividade espesiál ruma ba ema ne'ebé moras mentál, no sira nia família? Tanbasá presiza ka tanbasá la presiza? (Ezemplu mak fatin ba ema atu bá aprende buat ruma ka aumenta nia skill, klínika, sentru komunidade, nst)

**Konkluzaun**

1. Iha buat seluk ne'ebé Ita-Boot hakarak hato'o kona-ba ajuda/tratamentu ba saúde mentál iha [munisípiu/postu administrativu]?

## Guide 2: Decision maker interview guide

**Introduction**

Hello my name is Teresa Hall and I am a student from the University of Melbourne in Australia. I am here today to talk with you about what you think about mental health care in Timor-Leste. I am interested in what works and what can be done to strengthen the mental health system. You have agreed to take part in the interview and for me to audio record. It will take about one hour. Please let me know if you need a rest or want to stop the interview. Remember:

- Please tell me honestly what you think about mental health care.
- I will ask a series of questions and invite your response.
- There are no right or wrong answers. Your real experiences and thoughts are important.
- I won’t discuss what you tell me with anyone else; it is confidential.

**Service delivery and integration**

1. How effective are mental health services in Timor-Leste…? What are the strengths? Challenges? How can this be improved? Is this different at national, municipality and admin posts?
   1. delivering high quality care
   2. organised and structured
   3. linked to general health services
   4. linked to health care provided outside the health sector

*Prompt:* By NGOs? Social Solidarity? Education? Justice?

- 1. linked with customary healers? Should they be?

1. In your view, who should be involved in decision-making about treatment plans for people with mental illness? (Ask specifically about consumer and family involvement if not mentioned in response)
2. How do you think the unwell person and his/her family should be involved in decision-making about treatment (if at all)?
3. To what extent does the current approach to mental health care pay attention to the social aspects in the life of the unwell person (friends, family relationships, community participation, employment)? What are the barriers to this?
4. In the current health system, what would prevent service providers from involving the consumer and his/her family in treatment planning (lack of staff/resources, lack of training, lack of awareness, reluctance of consumers and their families)?

**Intersectoral collaboration: social network analysis measures**

1. Are you aware of [organisation] working in the mental health system?
2. Over the past year, how often have you had personal contact (e.g., meetings, phone calls, faxes, letters, or emails) with each of the following organisations?
   Response: (1) *no contact*; (2) *yearly*; (3) *quarterly*; (4) *monthly*; (5) *weekly*; (6) *daily.*
3. Over the past year, how often have you shared resources (e.g. funding, building space, transport, printing, materials) with each of the following organisations?
   Response: (1) *no resource sharing*; (2) *yearly*; (3) *quarterly*; (4) *monthly*; (5) *weekly*; (6) *daily.*

**Governance, participation and enabling environment**

The government of Timor-Leste makes decisions about what services are needed to help people who have a mental illness.

1. In Timor, how do people with mental illness generally find their way into the health system?
2. Are the following people/groups included in these decisions? Should they be? What makes this difficult? What would help this happen? *Prompt:* meetings, surveys, consultation
   1. people with mental illness
   2. their families
   3. organisations who work on behalf of people with mental illness
   4. service providers
   5. NGOs
   6. international organisations
   7. customary healers
   8. other

**Wrap**

1. Is there anything else you want to say about mental health in [municipality/admin post]?

Thank you very much for your participation

**Matadalan entrevista 2: ofisiál governu**

**Introdusaun**

Ha'u iha-ne'e ohin loron atu ko'alia ho Ita-Boot kona-ba tratamentu ne'ebé ema bele hetan ba moras (liuliu moras mentál nian) iha Timor-Leste. Ha'u buka informasaun kona-ba saida mak la'o di'ak, no buat ne'ebé la'o la di'ak, ka presiza atu hadi'a, tuir Ita-Boot nia hanoin, no tuir Ita-Boot nia esperiénsia. Objetivu husi peskiza ne'e mak atu hato'o informasaun ba governu no grupu seluk hodi bele halo sistema saúde mentál di'ak liu tan. Ita-Boot hatete ona ami katak Ita-Boot prontu atu foti parte iha peskiza ne'e, no prontu ba ha'u atu grava audio husi ita-nia entrevista ne'e. Entrevista ne'e sei han tempu maizumenus oras ida. Favór ida fó hatene ha'u se Ita-Boot presiza deskansa, ka hakarak para entrevista ne'e.

Informasaun adisionál kona-ba entrevista:

- Lalika ta'uk atu ko'alia buat ne'ebé tuir realidade, Ha'u hakarak rona buat ne'ebé di'ak, no mós buat ne'ebé la di'ak kona-ba sistema saúde ba moras mentál nian iha Timor-Leste.
- Ha'u sei husu pergunta, no Ita-Boot bele hatán konforme de'it Ita-Boot nia hakarak.
- Ita-Boot hatán saida de'it di'ak. Laiha resposta ne'ebé loos ka sala. Ita-Boot nia istória no hanoin mak importante.
- Ha'u nunka atu hatete ba ema seluk katak informasaun ne'ebé Ita-Boot dehan mai ha'u mai husi Ita-Boot. Bainhira peskiza ne'e hato'o rezultadu ba ema seluk, la uza ema nia naran.

**Tratamentu iha Timor-Leste**

1. Ajuda/tratamentu ne'ebé ema hetan ba moras mentál iha Timor-Leste efetivu ka lae? Saida mak di'ak kona-ba ajuda ne'ebé ema bele hetan ne'e? Nia dezafiu saida de'it? Tuir Ita-Boot nia hanoin, saida de'it mak bele hadi'a situasaun ne'e? Iha diferensa ka lae iha nivel nasionál, munisípiu no postu administrativu kona-ba asuntu hirak tuirmai ne'e?
   1. Fó ajuda/tratamentu ne'ebé ho kualidade di'ak ka lae?
   2. Organizadu no iha estrutura di'ak ka lae?
   3. Iha ligasaun ba servisu saúde jerál ka lae?.
   4. Iha ligasaun ba organizasaun hanesan PRADET, RHTO, Ministériu Edukasaun, Ministériu Solidariedade Sosiál ka organizasaun sira seluk ka lae?
   5. Iha ligasaun ba matan-dook ka lae? Tuir loloos, tenke iha ligasaun ba matan-dook ka lae?
2. Tuir ita-nia hanoin, sé de'it mak tenke envolve an bainhira halo planu ba tratamentu (*treatment plan*) ba ema ne'ebé moras mentál? (Husu espesifikamente kona-ba ema moras ne'ebé hetan ajuda ba nia moras ne'e, no ema ne'e nia família, se informasaun ne'e laiha iha resposta.)
3. Tuir Ita-Boot nia hanoin, ema ne'ebé moras tenke foti parte bainhira halo desizaun kona-ba ninia tratamentu ka lae? Se Ita-Boot sente nia tenke foti parte, nia foti parte ho maneira saida?
4. Agora daudaun iha Timor-Leste, tratamentu ba moras mentál haree situasaun sosiál ema moras nian ka lae? (Ezemplu- Ema moras nia kolega, relasaun família, partisipasaun iha komunidade, servisu?) Saida mak dezafiu ba ema atu halo/hetan tratamentu ne'ebé uza maneira ida ne'e?
5. Agora daudaun iha Timor-Leste, saida mak sei hapara doutór/a ka enfermeiru/a atu envolve ema moras no ninia família bainhira halo planu kona-ba ema moras nia tratamentu? (ezemplu mak ema ne'ebé hala'o servisu ne'e la to'o, rekursu ba tratamentu la to'o, treinamentu la to'o, koñesimentu kona-ba asuntu ne'e la to'o? Ema moras ka ema moras nia família lakohi?)

**Servisu Hamutuk**

1. Ita hatene kona-ba organizasaun saida de'it ne'ebé servisu iha sistema saúde mental?
2. Tinan ida liubá to'o agora, Ita-Boot kontaktu organizasaun hirak ne'e dala hira? (ezemplu: hasoru malu, ko'alia iha telefone, sms, mensajen iha facebook, mensajen iha whatsapp, hakerek ka simu surat, hakerek ka simu email?)
    (1) *laiha kontaktu*; (2) *tinan ida dala ida ka rua*; (3) *fulan tolu dala ida ka rua*; (4) *fulan ida dala ida ka rua*; (5) *semana ida dala ida ka rua*; (6) *loro-loron.*
3. “”, Ita-Boot fahe rekursu ba malu dala hira ho organizasaun hirak ne'e? (ezemplu: fundus, fatin servisu, transporte, print dokumentu, rekursu sira seluk) (1) *Nunka fahe rekursu ba malu*; (2) *tinan ida dala ida ka rua*; (3) *fulan tolu dala ida ka rua*; (4) *fulan ida dala ida ka rua*; (5) *semana ida dala ida ka rua*; (6) *loro-loron.*

**Sé mak halo desizaun?**

Governu Timor-Leste foti desizaun kona-ba ajuda saida de'it ne'ebé atu oferese ba povu Timor ne'ebé sofre ho moras mentál.

1. Normalmente, iha Timor-Leste, oinsá mak ema ne'ebé sofre ho moras mentál bele tama ba sistema saúde iha Timor-Leste atu hetan ajuda?
2. Ema/grupu ne'ebé tuirmai ne'e foti parte iha desizaun hirak ne'e ka lae? Sira loloos ne'e foti parte ka lae? Saida mak sei ajuda ne'e sai realidade? (Ezemplu: enkontru, survei, konsultasaun.)
   1. ema ho moras mental
   2. sira-nia família
   3. organizasaun advokasia
   4. Doutór/a no enfermeiru/a
   5. ONGs
   6. organizasaun internasionál
   7. matan-dook
   8. Seluk

**Konkluzaun**

1. Iha buat seluk ne'ebé Ita-Boot hakarak hato'o kona-ba ajuda/tratamentu ba saúde mentál iha [munisípiu/postu administrativu]?

## Guide 3: Service provider interview guide

**Introduction**

Hello my name is Teresa Hall and I am a student from the University of Melbourne in Australia. I am here today to talk with you about what you think about mental health care in Timor-Leste. I am interested in what works and what can be done to strengthen the mental health system. You have agreed to take part in the interview and for me to audio record it. It will take about one hour. Please let me know if you need a rest or want to stop the interview. Remember:

- Please tell me honestly what you think about mental health care.
- I will ask a series of questions and invite your response.
- There are no right or wrong answers. Your real experiences and thoughts are important.
- I won’t discuss what you tell me with anyone else; it is confidential.

**Service delivery**

1. What has been your experience of providing mental health care in Timor-Leste?
2. What are the positive aspects of your job providing mental health care?
3. What makes your job harder? Easier? How can this be improved?
4. In your view, who should be involved in decision-making about treatment plans for people with mental illness? (Ask specifically about consumer and family involvement if not mentioned in response)
5. How do you think the unwell person and his/her family should be involved in decision-making about treatment (if at all)?
6. To what extent does the current approach to mental health care pay attention to the social aspects in the life of the unwell person (friends, family relationships, community participation, employment)? What are the barriers to this?
7. In the current health system, what would prevent service providers from involving the consumer and his/her family in treatment planning (lack of staff/resources, lack of training, lack of awareness, reluctance of consumers and their families)?

**Intersectoral collaboration: social network analysis measures**

1. Are you aware of [organisation] working in the mental health system?
2. Over the past year, how often have you had personal contact (e.g., meetings, phone calls, faxes, letters, or emails) with each of the following organisations?
   Response: (1) *no contact*; (2) *yearly*; (3) *quarterly*; (4) *monthly*; (5) *weekly*; (6) *daily.*
3. Over the past year, how often have you shared resources (e.g. funding, building space, transport, printing, materials) with each of the following organisations?
   Response: (1) *no resource sharing*; (2) *yearly*; (3) *quarterly*; (4) *monthly*; (5) *weekly*; (6) *daily.*

**Governance, participation and enabling environment**

Your organisation makes decisions about how your service should operate to help people who have a mental illness.

1. In Timor, how do people with mental illness generally find their way into the health system?
2. Are the following people/groups included in these decisions? Should they be? What makes this difficult? What would help this happen? *Prompt:* meetings, surveys, consultation
   1. people with mental illness
   2. their families
   3. organisations who work on behalf of people with mental illness
   4. service providers
   5. customary healers
   6. other

**Wrap**

1. Is there anything else you want to say about mental health in [municipality/admin post]?

Thank you very much for your participation

**Matadalan entrevista 3: doutór/a ka enfermeiru/a**

**Introdusaun**

Ha'u iha-ne'e ohin loron atu ko'alia ho Ita-Boot kona-ba tratamentu ne'ebé ema bele hetan ba moras (liuliu moras mentál nian) iha Timor-Leste. Ha'u buka informasaun kona-ba saida mak la'o di'ak, no buat ne'ebé la'o la di'ak, ka presiza atu hadi'a, tuir Ita-Boot nia hanoin, no tuir Ita-Boot nia esperiénsia. Objetivu husi peskiza ne'e mak atu hato'o informasaun ba governu no grupu seluk hodi bele halo sistema saúde mentál di'ak liu tan. Ita-Boot hatete ona ami katak Ita-Boot prontu atu foti parte iha peskiza ne'e, no prontu ba ha'u atu grava audio husi ita-nia entrevista ne'e. Entrevista ne'e sei han tempu maizumenus oras ida. Favór ida fó hatene ha'u se Ita-Boot presiza deskansa, ka hakarak para entrevista ne'e.

Informasaun adisionál kona-ba entrevista:

- Lalika ta'uk atu ko'alia buat ne'ebé tuir realidade, Ha'u hakarak rona buat ne'ebé di'ak, no mós buat ne'ebé la di'ak kona-ba sistema saúde ba moras mentál nian iha Timor-Leste.
- Ha'u sei husu pergunta, no Ita-Boot bele hatán konforme de'it Ita-Boot nia hakarak.
- Ita-Boot hatán saida de'it di'ak. Laiha resposta ne'ebé loos ka sala. Ita-Boot nia istória no hanoin mak importante.
- Ha'u nunka atu hatete ba ema seluk katak informasaun ne'ebé Ita-Boot dehan mai ha'u mai husi Ita-Boot. Bainhira peskiza ne'e hato'o rezultadu ba ema seluk, la uza ema nia naran.

**Tratamentu iha Timor-Leste**

1. Ita-Boot nia esperiénsia fó ajuda/tratamentu ba ema ne'ebé sofre ho moras mentál iha Timor-Leste hanesan saida?
2. Aspetu pozitivu (buat ne'ebé di'ak) kona-ba Ita-Boot nia servisu {fó tratamentu/ajuda ba ema ne'ebé moras mentál) saida de'it?
3. Saida de'it mak halo Ita-Boot nia servisu susar liu? Saida de'it mak halo Ita-Boot nia servisu fasil liu?
4. Tuir ita-nia hanoin, sé de'it mak tenke envolve an bainhira halo planu ba tratamentu (*treatment plan*) ba ema ne'ebé moras mentál? (Husu espesifikamente kona-ba ema moras ne'ebé hetan ajuda ba nia moras ne'e, no ema ne'e nia família, se informasaun ne'e laiha iha resposta.)
5. Tuir Ita-Boot nia hanoin, ema ne'ebé moras tenke foti parte bainhira halo desizaun kona-ba ninia tratamentu ka lae? Se Ita-Boot sente nia tenke foti parte, nia foti parte ho maneira saida?
6. Agora daudaun iha Timor-Leste, tratamentu ba moras mentál haree situasaun sosiál ema moras nian ka lae? (Ezemplu- Ema moras nia kolega, relasaun família, partisipasaun iha komunidade, servisu?) Saida mak dezafiu ba ema atu halo/hetan tratamentu ne'ebé uza maneira ida ne'e?
7. Agora daudaun iha Timor-Leste, saida mak sei hapara doutór/a ka enfermeiru/a atu envolve ema moras no ninia família bainhira halo planu kona-ba ema moras nia tratamentu? (ezemplu mak ema ne'ebé hala'o servisu ne'e la to'o, rekursu ba tratamentu la to'o, treinamentu la to'o, koñesimentu kona-ba asuntu ne'e la to'o? Ema moras ka ema moras nia família lakohi?)

**Servisu Hamutuk**

1. Ita hatene kona-ba organizasaun saida de'it ne'ebé servisu iha sistema saúde mental?
2. Tinan ida liubá to'o agora, Ita-Boot kontaktu organizasaun hirak ne'e dala hira? (ezemplu: hasoru malu, ko'alia iha telefone, sms, mensajen iha facebook, mensajen iha whatsapp, hakerek ka simu surat, hakerek ka simu email?)
    (1) *laiha kontaktu*; (2) *tinan ida dala ida ka rua*; (3) *fulan tolu dala ida ka rua*; (4) *fulan ida dala ida ka rua*; (5) *semana ida dala ida ka rua*; (6) *loro-loron.*
3. “”, Ita-Boot fahe rekursu ba malu dala hira ho organizasaun hirak ne'e? (ezemplu: fundus, fatin servisu, transporte, print dokumentu, rekursu sira seluk) (1) *Nunka fahe rekursu ba malu*; (2) *tinan ida dala ida ka rua*; (3) *fulan tolu dala ida ka rua*; (4) *fulan ida dala ida ka rua*; (5) *semana ida dala ida ka rua*; (6) *loro-loron.*

**Sé mak halo desizaun?**

Governu Timor-Leste foti desizaun kona-ba ajuda saida de'it ne'ebé atu oferese ba povu Timor ne'ebé sofre ho moras mentál.

1. Normalmente, iha Timor-Leste, oinsá mak ema ne'ebé sofre ho moras mentál bele tama ba sistema saúde iha Timor-Leste atu hetan ajuda?
2. Ema/grupu ne'ebé tuirmai ne'e foti parte iha desizaun hirak ne'e ka lae? Sira loloos ne'e foti parte ka lae? Saida mak sei ajuda ne'e sai realidade? (Ezemplu: enkontru, survei, konsultasaun.)
   1. ema ho moras mental
   2. sira-nia família
   3. organizasaun advokasia
   4. Doutór/a no enfermeiru/a
   5. ONGs
   6. organizasaun internasionál
   7. matan-dook
   8. Seluk

**Konkluzaun**

1. Iha buat seluk ne'ebé Ita-Boot hakarak hato'o kona-ba ajuda/tratamentu ba saúde mentál iha [munisípiu/postu administrativu]?

## Guide 4: Civil society & Other community member/organisation interview guide

**Introduction**

Hello my name is Teresa Hall and I am a student from the University of Melbourne in Australia. I am here today to talk with you about what you think about mental health care in Timor-Leste. I am interested in what works and what can be done to strengthen the mental health system. You have agreed to take part in the interview and for me to audio record it. It will take about one hour. Please let me know if you need a rest or want to stop the interview. Remember:

- Please tell me honestly what you think about mental health care.
- I will ask a series of questions and invite your response.
- There are no right or wrong answers. Your real experiences and thoughts are important.
- I won’t discuss what you tell me with anyone else; it is confidential.

**Experience of care and service orientation**

1. What has been your experience with [government, NGO] mental health services?
2. Tell me about the mental health services in Timor-Leste…? What are the strengths? Challenges? How can this be improved?
   1. delivering high quality care
   2. organised and structured
   3. linked with customary healers? Should they be?
3. In your view, who should be involved in decision-making about treatment plans for people with mental illness? (Ask specifically about consumer and family involvement if not mentioned in response)
4. How do you think the unwell person and his/her family should be involved in decision-making about treatment (if at all)?
5. To what extent does the current approach to mental health care pay attention to the social aspects in the life of the unwell person (friends, family relationships, community participation, employment)? What are the barriers to this?
6. In the current health system, what would prevent service providers from involving the consumer and his/her family in treatment planning (lack of staff/resources, lack of training, lack of awareness, reluctance of consumers and their families)?

**Intersectoral collaboration: social network analysis measures**

1. Are you aware of [organisation] working in the mental health system?
2. Over the past year, how often have you had personal contact (e.g., meetings, phone calls, faxes, letters, or emails) with each of the following organisations?
   Response: (1) *no contact*; (2) *yearly*; (3) *quarterly*; (4) *monthly*; (5) *weekly*; (6) *daily.*
3. Over the past year, how often have you shared resources (e.g. funding, building space, transport, printing, materials) with each of the following organisations?
   Response: (1) *no resource sharing*; (2) *yearly*; (3) *quarterly*; (4) *monthly*; (5) *weekly*; (6) *daily.*

**Governance, participation and enabling environment**

The government of Timor-Leste makes decisions about what services are needed to help people who have a mental illness.

1. Are the following people/groups included in these decisions? Should they be? What makes this difficult? What would help this happen? *Prompt:* meetings, surveys, consultation
   1. people with mental illness
   2. their families
   3. organisations who work on behalf of people with mental illness
   4. service providers
   5. NGOs
   6. international organisations
   7. customary healers
   8. other

**Engagement, empowerment and participation**

1. How does the community currently learn about mental health (contributions of government and other organisations)? What would help to improve knowledge and understanding?
2. Does Timor-Leste need such an organisation? Why/not?

**Wrap**

1. Is there anything else you want to say about mental health in [municipality/admin post]?

Thank you very much for your participation.

**Matadalan entrevista 4: parte-interesadu/a seluk**

**Introdusaun**

Ha'u iha-ne'e ohin loron atu ko'alia ho Ita-Boot kona-ba tratamentu ne'ebé ema bele hetan ba moras (liuliu moras mentál nian) iha Timor-Leste. Ha'u buka informasaun kona-ba saida mak la'o di'ak, no buat ne'ebé la'o la di'ak, ka presiza atu hadi'a, tuir Ita-Boot nia hanoin, no tuir Ita-Boot nia esperiénsia. Objetivu husi peskiza ne'e mak atu hato'o informasaun ba governu no grupu seluk hodi bele halo sistema saúde mentál di'ak liu tan. Ita-Boot hatete ona ami katak Ita-Boot prontu atu foti parte iha peskiza ne'e, no prontu ba ha'u atu grava audio husi ita-nia entrevista ne'e. Entrevista ne'e sei han tempu maizumenus oras ida. Favór ida fó hatene ha'u se Ita-Boot presiza deskansa, ka hakarak para entrevista ne'e.

Informasaun adisionál kona-ba entrevista:

- Lalika ta'uk atu ko'alia buat ne'ebé tuir realidade, Ha'u hakarak rona buat ne'ebé di'ak, no mós buat ne'ebé la di'ak kona-ba sistema saúde ba moras mentál nian iha Timor-Leste.
- Ha'u sei husu pergunta, no Ita-Boot bele hatán konforme de'it Ita-Boot nia hakarak.
- Ita-Boot hatán saida de'it di'ak. Laiha resposta ne'ebé loos ka sala. Ita-Boot nia istória no hanoin mak importante.
- Ha'u nunka atu hatete ba ema seluk katak informasaun ne'ebé Ita-Boot dehan mai ha'u mai husi Ita-Boot. Bainhira peskiza ne'e hato'o rezultadu ba ema seluk, la uza ema nia naran.

**Tratamentu iha Timor-Leste**

1. Ita-Boot nia esperiénsia kona-ba ema ne'ebé sofre ho moras mentál iha Timor-Leste hanesan saida?
2. Ajuda/tratamentu ne'ebé ema hetan ba moras mentál iha Timor-Leste efetivu ka lae? Saida mak di'ak kona-ba ajuda ne'ebé ema bele hetan ne'e? Nia dezafiu saida de'it? Tuir Ita-Boot nia hanoin, saida de'it mak bele hadi'a situasaun ne'e? Iha diferensa ka lae iha nivel nasionál, munisípiu no postu administrativu kona-ba asuntu hirak tuirmai ne'e?
   1. Fó ajuda/tratamentu ne'ebé ho kualidade di'ak ka lae?
   2. Organizadu no iha estrutura di'ak ka lae?
   3. Iha ligasaun ba servisu saúde jerál ka lae?.
   4. Iha ligasaun ba organizasaun hanesan PRADET, RHTO, Ministériu Edukasaun, Ministériu Solidariedade Sosiál ka organizasaun sira seluk ka lae?
   5. Iha ligasaun ba matan-dook ka lae? Tuir loloos, tenke iha ligasaun ba matan-dook ka lae?
3. Tuir ita-nia hanoin, sé de'it mak tenke envolve an bainhira halo planu ba tratamentu (*treatment plan*) ba ema ne'ebé moras mentál? (Husu espesifikamente kona-ba ema moras ne'ebé hetan ajuda ba nia moras ne'e, no ema ne'e nia família, se informasaun ne'e laiha iha resposta.)
4. Tuir Ita-Boot nia hanoin, ema ne'ebé moras tenke foti parte bainhira halo desizaun kona-ba ninia tratamentu ka lae? Se Ita-Boot sente nia tenke foti parte, nia foti parte ho maneira saida?
5. Agora daudaun iha Timor-Leste, tratamentu ba moras mentál haree situasaun sosiál ema moras nian ka lae? (Ezemplu- Ema moras nia kolega, relasaun família, partisipasaun iha komunidade, servisu?) Saida mak dezafiu ba ema atu halo/hetan tratamentu ne'ebé uza maneira ida ne'e?
6. Agora daudaun iha Timor-Leste, saida mak sei hapara doutór/a ka enfermeiru/a atu envolve ema moras no ninia família bainhira halo planu kona-ba ema moras nia tratamentu? (ezemplu mak ema ne'ebé hala'o servisu ne'e la to'o, rekursu ba tratamentu la to'o, treinamentu la to'o, koñesimentu kona-ba asuntu ne'e la to'o? Ema moras ka ema moras nia família lakohi?)

**Servisu Hamutuk**

1. Ita hatene kona-ba organizasaun saida de'it ne'ebé servisu iha sistema saúde mental?
2. Tinan ida liubá to'o agora, Ita-Boot kontaktu organizasaun hirak ne'e dala hira? (ezemplu: hasoru malu, ko'alia iha telefone, sms, mensajen iha facebook, mensajen iha whatsapp, hakerek ka simu surat, hakerek ka simu email?)
    (1) *laiha kontaktu*; (2) *tinan ida dala ida ka rua*; (3) *fulan tolu dala ida ka rua*; (4) *fulan ida dala ida ka rua*; (5) *semana ida dala ida ka rua*; (6) *loro-loron.*
3. “”, Ita-Boot fahe rekursu ba malu dala hira ho organizasaun hirak ne'e? (ezemplu: fundus, fatin servisu, transporte, print dokumentu, rekursu sira seluk) (1) *Nunka fahe rekursu ba malu*; (2) *tinan ida dala ida ka rua*; (3) *fulan tolu dala ida ka rua*; (4) *fulan ida dala ida ka rua*; (5) *semana ida dala ida ka rua*; (6) *loro-loron..*

**Sé mak halo desizaun?**

Governu Timor-Leste foti desizaun kona-ba ajuda saida de'it ne'ebé atu oferese ba povu Timor ne'ebé sofre ho moras mentál.

1. Ema/grupu ne'ebé tuirmai ne'e foti parte iha desizaun hirak ne'e ka lae? Sira loloos ne'e foti parte ka lae? Saida mak sei ajuda ne'e sai realidade? (Ezemplu: enkontru, survei, konsultasaun.)
   1. ema ho moras mental
   2. sira-nia família
   3. organizasaun advokasia
   4. Doutór/a no enfermeiru/a
   5. ONGs
   6. organizasaun internasionál
   7. matan-dook
   8. Seluk

**Konkluzaun**

1. Iha buat seluk ne'ebé Ita-Boot hakarak hato'o kona-ba ajuda/tratamentu ba saúde mentál iha [munisípiu/postu administrativu]?
