# Supplementary material for: Intersectoral collaboration for people-centred mental health care in Timor-Leste: a mixed-methods study using qualitative and social network analysis
Source: Int J Ment Health Syst. 2019 Nov 16;13:72. doi: 10.1186/s13033-019-0328-1 (PMC6858633; doi:10.1186/s13033-019-0328-1)
Supplement: Supplementary file 2 — Additional file 2: Table S1. Summary of key government strategies and law pertaining to mental health in Timor-Leste. [file 13033_2019_328_MOESM2_ESM.docx]

# Appendix Table S1: summary of key government strategies and law pertaining to mental health in Timor-Leste

| Data source and primary responsible stakeholder | Key points relevant to mental health |
| --- | --- |
| National Mental Health Strategy 2018-2022 (1); Ministry of Health | Objectives   1. “Scale up human resources for mental health 2. Strengthen leadership and governance for a multi-sectoral response to mental health 3. Increase promotion and prevention strategies for mental health 4. Provide comprehensive community-based mental health and social services 5. Ensure access to necessary infrastructure and equipment for service delivery 6. Improve information systems, monitoring and evaluation, research and evidence for mental health; 7. Mobilise resources to effectively finance mental health care”   Specific to intersectoral action and participatory governance:   1. MoH to work with all relevant sectors to ensure that mental health is integrated and implemented according to human rights instruments including UNCRPD 2. MoH to engage stakeholders from all relevant sectors, including persons with mental disorders, carers/family members and informal sectors, in the development and implementation of policies, laws and services relating to mental health 3. MoH to establish mechanisms to formalise participation of people with mental illness and psychosocial disabilities in health policy, law and services. |
| National Health Sector Strategic Plan 2011-2030 (2); Ministry of Health | Objectives:   1. Strengthen the stewardship role of Ministry of Health 2. Ensure access to quality primary, secondary and tertiary health care, especially for vulnerable groups (i.e. children, women, others) 3. Bolster human resources 4. Deliver sufficient health infrastructure including facilities, medical equipment, transport, data management 5. Strengthen health administration and management.   Specific to mental health:   - Mental health is a crucial component of primary health care - Recognise the marginalisation and stigmatisation of people with mental illness |
| National Strategy for Prevention and Control of Noncommunicable Diseases (NCDs), Injuries, Disabilities and Care of the Elderly & NCD National Action Plan 2014-2018. (3); Ministry of Health | Objectives:   1. Lead multi-sectoral partnerships 2. Create healthy environments to promote health and reduce the risk of development of NCDs 3. Improve access to health care services and NCD-literacy of health professionals 4. Establish a sustainable surveillance, monitoring and evaluation system   Specific to mental health:   - Increase availability of clinical psychologists. |
| Comprehensive service package for primary health care (4); Ministry of Health | Objectives   1. To provide comprehensive and integrated Primary Health Care services; 2. To enhance the access, improve equity, quality and efficiency of health services; 3. To meet the diverse, simple and complex health needs of the population; and 4. Using primary health care as the means to extend health care to the entire population   Summary of items in policy relating to mental health:   - Mental health care is provided through the primary health care programme - People with disabilities and chronic health conditions are identified as priority population groups. - Each health post (suku level) is tasked with providing assistance to families affected by mental illness; mental health care is provided by the administrative post and municipality health staff - Family is the unit of the community at which interventions are aimed - Mental illness and disability are referred to as separate categories throughout the document   Mental health components of package to control and prevent NCDs:   - Mental health and epilepsy promotion; Home visits; Counselling to people with mental illness and epilepsy and their families; Diagnosis and treatment; and provide community-based rehabilitation |
| National Policy for Inclusion and Promotion of the Rights of People with Disabilities (5); Coordinated by the Ministry of Social Solidarity; coordination with 7 partner ministries and secretaries | Objectives:   1. Promote equal opportunities, active participation and improving the quality of life for people with disabilities. 2. Define areas of and strategies for prevention, treatment, rehabilitation and integration of the people with disabilities. 3. Create a mechanism of cooperation and coordination for intersectoral action   Summary   - Includes people with temporary or permanent ‘mental inability’ - Policy specifies strategies for: health, education, vocational training and employment, social assistance, justice, culture and sports, Accessibility and Mobility, Information and Communication, and Gender Equality. - Specifies 11 strategies to increase health access for people with disabilities, including: dissemination of information about disability; early intervention activities for children with disabilities; provide holistic physical and mental rehabilitation services; secure the issuing of medical statements certifying the temporary or permanent disability of the disabled person; increase specialised human resources for disability. |
| National Action Plan for People with Disabilities 2014-2018 (unapproved but unofficially referred to); Ministry of Social Solidarity; coordination with 10 partner ministries and secretaries | - Provides 10 sector action plans for relevant ministries/secretaries to include in implementation of existing and future action plans: (1) education; (2) employment and vocational training; (3) gender equality; (4) justice; (5) health; (6) information and communication; (7) accessibility and mobility, public works; (8) transport and communication; (9) sport and culture; and (10) social assistance.   Specific to mental health:   - Development of health professionals with specialized mental health training including psychiatrists, psychologists, psychiatric nurses, social workers, occupational therapists |
| Timor-Leste Strategic Development Plan 2011-2030 (6); Government of the Democratic Republic of Timor-Leste | Objectives for Health:   1. Ensure quality primary health care services are accessible to all Timorese 2. Focus on the needs of children, women and other vulnerable groups 3. Develop a hospital service, including specialist care.   Specific to mental health:   - “Improve access to health facilities and treatment for all people with mental illness or epilepsy - Provide acute care facilities at referral hospitals for mental health patients - Introduce a comprehensive multi-disciplinary team of psychiatrists, psychiatric nurses, psychologists and mental health technical professionals who are appropriately skilled and have reached specific standards of training - Increase community awareness and understanding of mental illness and epilepsy through advocacy, education and promotion”.   Specific to vulnerable families   - Includes strategies to protect vulnerable families, including people with disabilities - Rehabilitation and reintegration of prisoners - Create employment, income and training opportunities for vulnerable families - Ensure access to education |
| National Policy on Inclusive Education 2016 (7); Ministry of Education | Specific to mental health:   - Promote the physical and psychosocial health of teachers and pupils, especially vulnerable children - Support for children's psychological and emotional health by combatting bullying and corporal punishment - Develop standards for inclusive education and tailor teaching and schools to meet the age, gender, psychosocial needs of vulnerable students including, children with disabilities, out-of-school children, unsuccessful students; |
| Timor-Leste Constitution (8); Government of the Democratic Republic of Timor-Leste | - Section 16: General principles. Specifies non-discrimination on the basis colour, race, marital status, gender, ethnical origin, language, social or economic status, political or ideological convictions, religion, education and physical or mental condition. - Section 21: Disabled citizens. Promotion and protection of the rights and duties of persons with disabilities, except for the rights and duties which the person with disability is unable to exercise or fulfil due to their disability. - Section 32: Limits on sentences and security measure. States that in case of danger as a result of mental illness, security measures may be extended successively by judicial decision |
| Justice Sector Strategic Plan for Timor-Leste 2011-2030 (9); Council of Coordination for Justice (i.e. Minister of Justice, President of the Court of Appeal and Prosecutor General) | - General principles specify recognition of all citizens equally before the law and grant them the same rights and obligations, and non-discrimination on the basis of race, gender, civil status, ethnic origin, language, social status or economic situation, political and religious convictions, education or physical and mental condition. - Specific mention to increase access to the formal justice sector for ‘disadvantaged and vulnerable groups’ (women and children named). - Specifically identifies a challenge to prison implementation as improving health and mental health to detainees, particularly those with mental illness |

# References

1. Ministry of Health Timor-Leste. National Mental Health Strategy 2018-2022: for a Mentally Healthy Timor-Leste. Dili, Timor-Leste; 2018.

2. Ministry of Health Timor Leste. National Health Sector Strategic Plan 2011-2030. Dili: Government of the Democratic Republic of Timor Leste; 2011.

3. Ministry of Health Timor Leste. National Strategy for Prevention and Control of Noncommunicable Diseases (NCDs), Injuries, disabilities and care of the elderly & NCD National Action Plan 2014-2018. Dili, Timor-Leste: Ministry of Health; 2014.

4. Ministry of Health Timor Leste. Comprehensive service package for primary health care. Dili: Government of the Democratic Republic of Timor Leste; 2015.

5. Government of the Democratic Republic of Timor Leste. National Policy for Inclusion and Promotion of the Rights of People with Disabilities. Dili, Timor-Leste: Government of the Democratic Republic of Timor Leste; 2012.

6. Timor-Leste National Parliament. Timor-Leste Strategic Development Plan 2011-2030. Dili, Timor-Leste: Timor-Leste National Government; 2011.

7. Ministry of Education Timor-Leste. National Policy on Inclusive Education. Dili, Timor-Leste: Ministry of Education; 2016.

8. Constitution of Democratic Republic of Timor-Leste, (2002).

9. Council of Coordination for Justice. Justice Sector Strategic Plan for Timor-Leste 2011-2030 Dili, Timor-Leste: Council of Coordination for Justice; 2010.
